# Gene Therapy in a Mouse Model of Niemann–Pick Disease Type C1

**DOI:** 10.1089/hum.2020.175

**Published:** 2021-06-16

**Authors:** Yoshie Kurokawa, Hitoshi Osaka, Takeshi Kouga, Eriko Jimbo, Kazuhiro Muramatsu, Sachie Nakamura, Yuki Takayanagi, Tatsushi Onaka, Shin-ichi Muramatsu, Takanori Yamagata

**Affiliations:** ^1^ Department of Pediatrics, Jichi Medical University, Tochigi, Japan.; ^2^ Division of Brain and Neurophysiology, Department of Physiology, Jichi Medical University, Tochigi, Japan.; ^3^ Division of Neurological Gene Therapy, Center for Open Innovation, Jichi Medical University, Tochigi, Japan.; ^4^ Center for Gene and Cell Therapy, The Institute of Medical Science, The University of Tokyo, Tokyo, Japan.

**Keywords:** Niemann–Pick disease type C1, gene therapy, AAV vector, intracisternal injection

## Abstract

Niemann–Pick disease type C1 (NPC1) is a fatal congenital neurodegenerative disorder caused by mutations in the *NPC1* gene, which is involved in cholesterol transport in lysosomes. Broad clinical manifestations of NPC1 include liver failure, pulmonary disorder, neurological deficits, and psychiatric symptoms. The main cause of death in NPC1 patients involves central nervous system (CNS) dysfunction; there is no essential treatment. We generated a tyrosine-mutant adeno-associated virus (AAV) 9/3 vector that expresses human *NPC1* under a cytomegalovirus (CMV) promoter (AAV-CMV-*hNPC1*) and injected it into the left lateral ventricle (5 μL) and cisterna magna (10 μL) of *Npc1* homo-knockout (*Npc1*^−*/*−^) mice. Each mouse received total 1.35 × 10^11^ vector genome on days 4 or 5 of life. AAV-treated *Npc1*^−*/*−^ mice (*n* = 11) had an average survival of >28 weeks, while all saline-treated *Npc1*^−*/*−^ mice (*n* = 11) and untreated *Npc1*^−*/*−^ mice (*n* = 6) died within 16 weeks. Saline-treated and untreated *Npc1*^−*/*−^ mice lost body weight from 7 weeks until death. However, the average body weight of AAV-treated *Npc1*^−*/*−^ mice increased until 15 weeks. AAV-treated *Npc1*^−*/*−^ mice also showed a significant improvement in the rotarod test performance. A pathological analysis at 11 weeks showed that cerebellar Purkinje cells were preserved in AAV-treated *Npc1*^−*/*−^ mice. In contrast, untreated *Npc1*^−*/*−^ mice showed an almost total loss of cerebellar Purkinje cells. Combined injection into both the lateral ventricle and cisterna magna achieved broader delivery of the vector to the CNS, leading to better outcomes than noted in previous reports, with injection into the lateral ventricles or veins alone. In AAV-treated *Npc1*^−*/*−^ mice, vector genome DNA was detected widely in the CNS and liver. Human *NPC1* RNA was detected in the brain, liver, lung, and heart. Accumulated unesterified cholesterol in the liver was reduced in the AAV-treated *Npc1*^−*/*−^ mice. Our results suggest the feasibility of gene therapy for patients with NPC1.

## INTRODUCTION

Niemann–Pick disease type C1 (NPC1) [OMIM:257220] is an autosomal recessive lipid storage disorder caused by mutations in the *NPC1* gene located in chromosome 18q11.^1^ The incidence of NPC1 is reported to exceed 1:100,000,^2^ and more than 300 pathogenic mutations of this gene have been reported.^3,4^ As NPC1 plays a central role in transporting cholesterol from late endosomes or lysosomes into the membrane,^5^ dysfunction of NPC causes the accumulation of cholesterol and some other sphingolipids, such as sphingomyelin and glycosphingolipids in cells, which leads to the degeneration of neurons in the central nervous system (CNS), particularly the loss of Purkinje cells in the cerebellum.^6^

Although NPC1 is a systemic progressive disease, the main symptoms are related to the dysfunction of the CNS and liver.^7^ Characteristic neurological symptoms are severe developmental delay, especially in early infantile type, and deterioration with vertical supranuclear gaze palsy and cataplexy in the late infantile to adult types. Other symptoms include hypotonia, cerebellar ataxia, dystonia, epileptic seizures, and psychiatric problems.^8^

NPC1 is suspected based on clinical symptoms, such as severe liver dysfunction with hepatosplenomegaly in infancy and neurological symptoms. The NPC Suspicion Index is commonly used in the clinical setting.^9^ A bone marrow examination reveals foamy cells that are leukocytes accumulating cholesterol. Filipin staining is used to detect the accumulation of unesterified cholesterol in leukocytes or fibroblasts. A definite diagnosis of NPC1 is made by a genetic analysis.^10^ The pathological examination shows the swelling of neurons and axons throughout the brain, particularly in the cerebral and cerebellar white matter, brain stem, and posterior columns of the spinal cord.^11^ In the end stage of the disease, neuronal cell death and severe demyelination occur.^12^

The only drug approved for treatment, miglustat, is a glucosylceramide synthase inhibitor that can only delay disease progression.^13^ Several other therapies have been investigated, such as that with the histone deacetylase inhibitor vorinostat,^14^ which strengthens the function of molecular chaperones and improves the folding of mutant NPC protein in the endoplasmic reticulum, and 2-hydroxypropyl-β-cyclodextrin (HPβCD),^15,16^ which improves the transport kinetics of cholesterol in the membrane. Combined therapy with cyclodextrin/allopregnanolone and miglustat has been reported to ameliorate the motor functions with an increased number of neurons in the cerebellum.^17,18^ However, all of these therapies showed only marginal effects.

Recently, gene therapy with adeno-associated virus (AAV) 9 vector was applied to a mouse model of NPC1.^19–21^ In those previous studies, the injection route of the vector was either systemic or intracerebroventricular. The promoters used in those studies were cytomegalovirus (CMV), calcium/calmodulin-dependent protein kinase II (CaMK II), elongation factor (EF1α), and synapsin I (Syn I). These gene therapies showed the amelioration of neurodegeneration and improvements in behavior analyses, but the life span expansion was still limited. To obtain better effects, the transduction of broad areas in the CNS is important.

We herein report our investigation of the effect of combined injection into both the left lateral ventricle and cisterna magna in *Npc1* knockout mice.

## MATERIALS AND METHODS

### Production of recombinant AAV vectors

**Figure 1.**
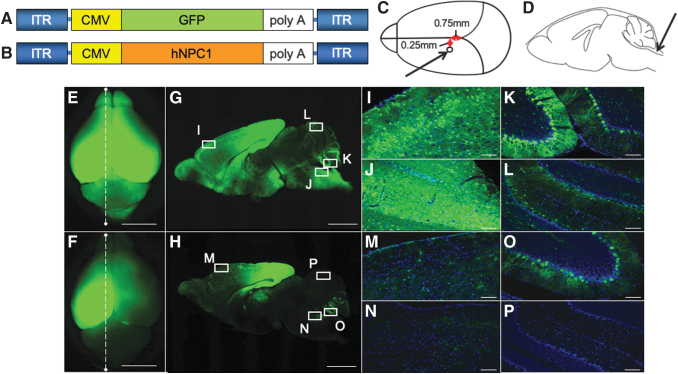
Broad transduction of the brain by AAV-GFP vector. We generated the tyrosine-mutant AAV9/3 vector with CMV promoter. **(A)**, **(B)** An illustration of the vector constructs. The expression cassette consisting of CMV promoter, cDNA of *GFP* or *hNPC1*, and simian virus (SV) 40 poly A were inserted between the inverted terminal repeat (ITR) of AAV3. Capsids were tyrosine-mutant AAV9. The vectors were prepared at a titer of 1.8 × 10^10^ vg/μL. **(C)** Five microliters of twofold diluted AAV-GFP vector was injected into the left ventricle. The stereotaxic coordination was 0.75 mm anterior from the lambdoid suture and 0.25 mm *left* from the *center*, and 2 mm deep from the surface of the skull. **(D)** Ten microliters of AAV-GFP was injected into the cisterna magna. The total injection dosage was 15 μL (1.35 × 10^11^ vg) per mouse. *Arrows* indicate the positions of the injection. A distribution analysis of the GFP vector in the brain at 3 weeks after the injection of AAV-GFP vector. E and F are whole-brain GFP fluorescent images obtained by a stereomicroscope. G and H are sagittal sections of E and F, respectively. **(E, G)** Both cerebral hemispheres showed strong signals. Milder signals were observed in the cerebellum. **(F, H)** In this mouse, we injected 5 μL of AAV-GFP vector only into the left ventricle. Strong signals in the left hemisphere and slight signals in the right hemisphere were observed, but fewer signals were noted in the cerebellum. Higher magnification of the frontal cortex **(I, M)**, brain stem **(J, N)**, lower part of the cerebellum **(K, O)**, and upper part of the cerebellum **(L, P)** was shown. Strong signals were detected in I, J, and K, compared with M, N, and O. In L, the signal was weakly observed. Scale bar = 5 mm **(E, F)**, 2 mm **(G, H)**, 100 μm **(I–P)**. AAV-GFP, adeno-associated virus-green florescent protein; CMV, cytomegalovirus; hNPC1, human Niemann–Pick type C1 gene.

AAV vector plasmid contained an expression cassette consisting of the CMV immediate-early promoter and cDNA of green florescent protein (*GFP*) (Fig. 1A) or human *NPC1* (GenBank, AB209048.1) (Fig. 1B) and the simian virus 40 polyadenylation signal sequence between the inverted terminal repeats of the AAV3 genome. AAV9 vp cDNA was synthesized, and the sequence was identical to that previously described,^22^ except for the substitution of thymidine for adenine 1337, which introduces an amino acid change from tyrosine to phenylalanine at position 446.^23^ Recombinant AAV vectors were produced by transient transfection of HEK293 cells using the vector plasmid, an AAV3 rep, the tyrosine-mutant AAV9 vp expression plasmid, and the adenoviral helper plasmid pHelper (Agilent Technologies, Santa Clara, CA), as described previously.^24^ The recombinant viruses were purified by isolation from two sequential continuous CsCl gradients, and the viral titers were determined by quantitative PCR (qPCR).

### *Npc1* deficient mice and genotyping

We ordered FVB.C-*Npc1*<M1N>/J (Stock#021755) mice from the Jackson Laboratory (Bar Harbor, ME) and reproduced them by crossing heterotypes. DNA samples were extracted from the tip of mouse tails at 2 to 3 days after birth and were used for PCR, as follows: 47 cycles at 95°C for 30 s for denaturing, at 58°C for 45 s for annealing, and at 72°C for 45 s for extension. The primers used to detect the wild type were forward 5′-GGTGCTGGACAGCCAAGTA-3′ and reverse 5′-CGATGGCAGATGAGCTACAG-3′, and the forward primer for the mutant type was the same as that for the wild type, while the reverse primer was 5′-TGAGCCCAAGCATAACTTCC-3′ (data not shown).

Mice were fed standard chow for mice. In addition, some *Npc1*^−*/*−^ mice and treated mice were fed gel-type food (DietGel^®︎^ 76A and DietGel^®^ Recovery; ClearH_2_O^®^, Portland, ME) from week 9, which is when *Npc1*^−*/*−^ mice have difficulty eating regular solid food. We divided mice into five groups, as follows: AAV-treated *Npc1^+/+^* mice fed gel food (group a); AAV-treated *Npc1*^−*/*−^ mice fed gel food (group b); saline-treated *Npc1*^−*/*−^ mice fed gel food (group c); untreated *Npc1*^−*/*−^ mice fed gel food (group d); and untreated *Npc1*^−*/*−^ mice fed standard food (group e), with 11, 12, 11, 6, and 6 mice in each group, respectively (Supplementary Table S1).

### Establishment of the AAV-*hNPC1* vector injection route

We analyzed the distribution of the vector to establish a better route for delivering AAV vector to the whole brain, including the cerebellum. First, we injected tyrosine-mutant AAV9/3-CMV-GFP vector into the left lateral ventricle of mice on days 4 to 5 after birth. The injection point was set at 0.75 mm toward the nose from the lambdoid suture, 0.25 mm left of the midline, and 2 mm deep (Fig. 1C).^25^ The amount of AAV-GFP was 5 μL. Next, we injected the vector into the cisterna magna just below the most inferior point of the cranial bone and 2 mm deep (Fig. 1D).^26^ The amount of AAV-GFP was 10 μL. We injected AAV-GFP into both the left ventricle and cisterna magna for six *Npc1*^−*/*−^ mice, and only into the left ventricle for three *Npc1*^−*/*−^ mice to analyze the difference in distribution. At 3 weeks after the injection, we sacrificed the mice and detected the expression of GFP by a Leica M165 FC stereomicroscope (Leica, Wetzlar, Germany) (Fig. 1E, F). We then fixed the brains with 4% paraformaldehyde and transferred them into 15% sucrose, followed by 30% sucrose, allowing 3 days for each step. The brains were frozen after being embedded in O.C.T. compound (Sakura Finetek Co., Ltd., Tokyo, Japan) and cut into 30-μm sections, and then GFP was detected (Fig. 1G, H). In Fig. I–P, images were merged with Hoechst staining (Dojindo Molecular Technologies, Kumamoto, Japan) and observed under a FluoView™ FV1000 confocal microscope (Olympus, Tokyo, Japan).

### AAV-*hNPC1* vector injection and the comparison of the phenotype with controls

We injected mice with AAV-*hNPC1* at days 4 to 5 after birth, before the fur had covered the animals' heads. The injection sites were described in the previous section and are shown in Fig. 1C, D. The control mice were injected with the same saline solution mentioned before. We used cooling anesthesia for the injection, placing mice on a plastic sheet in contact with ice until they stopped moving and then transferring them to the injection instrument. After injection, we placed the animals on a hot plate at 37°C and then returned them to their mothers.

### The analysis of vector genome quantity by qPCR after AAV-*hNPC1* injection

At 10 weeks after AAV-*hNPC1* injection, we sacrificed the mice under CO_2_ anesthesia and resected their tissues. We extracted genomic DNA from the brain, cerebellum, brain stem, spinal cord, and liver using a DNeasy Blood & Tissue Kit (QIAGEN, Venlo, The Netherlands). We performed qPCR using 100 ng of genomic DNA. To quantify the AAV vector genome, we drew a standard curve using 1 μL AAV-*hNPC1* solutions ranging in concentration from 10^2^ to 10^7^ vg/μL as a template. According to the manufacturer's protocol, we performed quantitative real-time PCR using an Applied Biosystems^®^ 7500 Fast Real-Time PCR System (Applied Biosystems, Life Technologies, Carlsbad, CA) with 2 × TaqMan^®^ Fast Universal PCR Master Mix (Thermo Fisher Scientific, Waltham, MA), genomic DNA, primers, and a dual fluorescently labeled (FAM/MGB) probe according to the specific sequence of AAV-*hNPC1*. The sequences of the primers and probe are listed in Supplementary Table S2.

**Figure 2.**
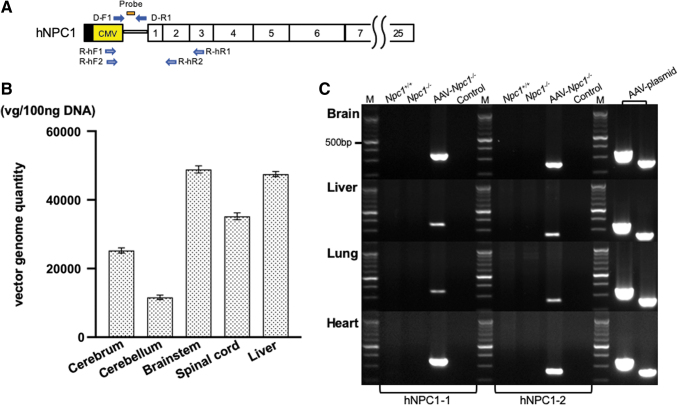
Vector genome distribution and expression of *hNPC1*. **(A)** Location of the primers and probe. The open vertical rectangles represent coding exons. The vector genome DNA was amplified using the primer set of D-F1 and D-R1. The *hNPC1* mRNA was amplified using the two primer sets of R-hF and R-hR. **(B)** Quantitation of the vector genome by qPCR. The vector genome was detected in the broad area of CNS, including the brain stem and spinal cord. It was also detected in the liver. **(C)** Detection of transgene-specific mRNA in the brain, liver, lung, and heart. The expression of transgene-specific *hNPC1* was detected only in the AAV-treated *Npc1*^−*/*−^ mice, whereas no fragments were amplified in the untreated *Npc1^+/+^* or *Npc1*^−*/*−^ mice. We used the AAV9/3-CMV-*hNPC1* plasmid as a positive control. vg/100 ng DNA, vector genomes per 100 ng DNA; M, molecular marker; Control, control without template cDNA. CNS, central nervous system; qPCR, quantitative PCR.

The positions of the probe and primers in AAV-*hNPC1* are indicated in Fig. 2A.

The PCR conditions were as follows: 1 cycle at 95°C for 20 s, and 40 cycles at 95°C for 3 s and 60°C for 30 s. We performed each experiment in triplicate and presented the data as the mean ± standard error of the mean.

### The analysis of the exogenous *hNPC1* and endogenous *mNpc1* mRNA expression by reverse transcription-PCR

To confirm the expression of exogenous human *NPC1* and murine *Npc1,* we performed reverse transcription (RT)-PCR using the brain, liver, lung, and heart from untreated *Npc1^+/+^*, *Npc1*^−*/*−^, and AAV-treated *Npc1*^−*/*−^ mice 10 weeks after AAV-*hNPC1* injection. RT-PCR using total RNA extracted from mouse tissues was performed as previously described.^27^ In brief, total RNA extracted via the manual protocol was subjected to reverse transcription, and 1 μL of cDNA was used for PCR followed by PCR amplification using TaKaRa rTaq™ DNA Polymerase (TaKaRa Bio, Inc., Otsu, Japan).

We performed RT-PCR with each primer (Fig. 2A, C, Supplementary Fig. S1, and Supplementary Table S2) under the following PCR conditions: 94°C for 2 min, 30 cycles at 94°C for 15 s, 62 or 64°C for 30 s, and 72°C for 1 min with additional elongation at 72°C for 7 min. As an internal control, the expression of murine glyceraldehyde-3-phosphate dehydrogenase (GAPDH) was also measured in each sample under the same PCR conditions.

Cerebral cortex was used as the brain. After obtaining the results of electrophoresis, we analyzed the signal intensity of the bands compared with the positive control (AAV-plasmid) DNA for each organ by a Gel Doc EZ Imager (Bio-Rad, Hercules, CA).

### The analysis of the survival by a Kaplan–Meier curve and body weight changes

The survival of each mouse was plotted on a Kaplan–Meier curve. The body weight of each mouse was measured once a week after 5 weeks and plotted on the graph.

### Rotarod test

To compare the motor function in each group, we performed the rotarod test. Mice were placed on an accelerating wheel (Rotarod; O'HARA & CO., LTD., Tokyo, Japan), and the time it took for them to fall from the rod was measured. The rotating bar was 30 mm in diameter and rotated at 4–40 rpm, just as previously reported.^28^

### Histological analyses

At 11 weeks of age, mice were sacrificed under CO_2_ anesthesia, and their tissues were resected. These were fixed by 4% paraformaldehyde and placed into 15% sucrose, followed by 30% sucrose, allowing 3 days for each step. Tissues were frozen after being embedded in O.C.T. compound (Sakura Finetek). We cut the brain into 30-μm-thick sections and used the following antibodies for histological analyses: anti-calbindin antibody (c9848; Sigma-Aldrich Co., St. Louis, MO) at 1:1,500 as the primary antibody and goat anti-mouse IgG H&L Alexa Fluor^®^ 594 (ab150116; Abcam) at 1:300 as the secondary antibody. We then obtained their images with a BZ-X810 fluorescence microscope (Keyence, Osaka, Japan) and confocal images with a FluoView FV1000 confocal microscope (Olympus).

We also cut the livers into 10-μm-thick sections for filipin (F9765; Sigma-Aldrich Co.) staining. After washing the liver with phosphate-buffered saline (PBS) three times, we incubated the tissue with 1.5 mg/mL glycine/PBS at room temperature for 10 min and then performed staining with filipin working solution in the dark for 2 h. The livers were examined and imaged after being washed three times in PBS under a BZ-X810 fluorescence microscope.

### Statistical analyses

The GraphPad Prism 8 software program (GraphPad Software, San Diego, CA) was used to generate graphs and to statistically analyze the vector genome DNA qPCR, survival, and rotarod test data. We determined the survival ratio using the Kaplan–Meier curve. We used Wilcoxon signed-rank test to compare the average time spent on the rotarod among the three groups.

### Animal experiments

All animal studies were approved by the Animal Care Committee, Jichi Medical University (approval number, 17213-01)

## RESULTS

### The GFP expression of brain after AAV-GFP vector injection

The gross distribution of GFP was observed by a stereomicroscope. All six *NPC1*^−*/*−^ mice with AAV-GFP injected into both the left lateral ventricle and cisterna magna showed the GFP expression in the whole brain, including the cerebellum (Fig. 1E). In contrast, three *NPC1*^−*/*−^ mice with AAV-GFP injected into left lateral ventricle alone showed the GFP expression in only the middle part of the left cerebral hemisphere (Fig. 1F).

Histological analyses confirmed the broad expression after the combined injection into the lateral ventricle and cisterna magna. In sagittal sections of whole brain after the combined injection, strong signals were detected in the cerebrum and milder signals in the cerebellum (Fig. 1G). Strong signals were detected in the frontal cortex (Fig. 1I), brain stem (Fig. 1J), and the lower part of the cerebellum (Fig. 1K); however, the upper part of the cerebellum showed a weaker signal (Fig. 1L). On the contrary, only the parietal lobe showed a high signal in mice with injection into the ventricle (Fig. 1H).

### The detection of vector genome DNA

Vector genome was detected in the broad areas of the CNS, especially the brain stem (mean vector genome per 100 ng DNA: 48867.2) and spinal cord (35218.5). The same amount of vector genome was detected in the liver (47525.2). It was also detected in the cerebrum (25265.9), while a lower amount was detected in the cerebellum (11623.4).

### The expression of *hNPC1* and *mNpc1*

We prepared two sets of *hNPC1* primers—*hNPC1*–1 (R-hF1 and R-hR1) and *hNPC1*–2 (R-hF2 and R-hR2)—to detect the expression of AAV-*hNPC1* vector. Both sets detected the expression of *hNPC1* in AAV-treated *Npc1*^−*/*−^, but not in untreated *Npc1^+/+^* and *Npc1*^−*/*−^ in the brain (Fig. 2C). In addition, *hNPC1* was also detected in the liver, lung, and heart. We then analyzed the expression ratio of *hNPC1* RNA in the brain, liver, lung, and heart toward the AAV-plasmid. The first *hNPC1* primer set showed brain, liver, lung, and heart ratios of 0.31, 0.09, 0.05, and 0.50, while the second *hNPC1* primer set showed ratios of 0.45, 0.16, 0.09, and 0.55, respectively (Table 1). The expression of murine *Npc1* was detected in untreated *Npc1^+/+^*, but not in untreated *Npc1*^−*/*−^ or AAV-treated *Npc1*^−*/*−^ (Supplementary Fig. S1).

**Table 1.** The results of an expression analysis of hNPC1 RNA

Tissue/AAV-Plasmid	Primer	
	hNPC1–1	hNPC1–2
Brain	0.31	0.45
Liver	0.09	0.16
Lung	0.05	0.09
Heart	0.50	0.55

hNPC1, human Niemann–Pick type C1.

### Survival analyses

**Figure 3.**
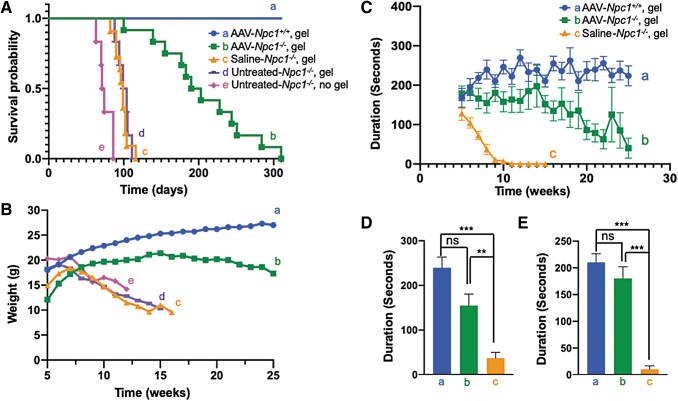
Improvement of life span, body weight loss, and rotarod performance in AAV-*Npc1*^−*/*−^ mice. **(A)** Kaplan–Meier survival curve. The average life span of untreated-*Npc1*^−*/*−^ without gel feeding was 75 days (63–86 days), but both untreated and saline-treated *Npc1*^−*/*−^ with gel feeding survived for an average of 100 days (88–111 days) and 98 days (82–116 days), respectively. The average life span was prolonged by ∼25 days simply by gel feeding. AAV-treated *Npc1*^−*/*−^ mice lived for 205 days (100–310 days) on average, which was 105 days longer than untreated and saline-treated *Npc1*^−*/*−^ mice with gel feeding. The maximum life span of AAV-treated *Npc1*^−*/*−^ reached 310 days. **(B)** Body weight changes. We assessed the body weight of every mouse weekly and plotted the average of each group from weeks 5 to 25. However, there were marked differences between the groups at the starting point. All three untreated *Npc1*^−*/*−^ mice groups lost body weight rapidly from weeks 5 to 7 until they died, whereas the AAV-treated *Npc1*^−*/*−^ mice showed a body weight gain until week 15 and thereafter showed a slight body weight loss. **(C–E)** The rotarod performance. We compared the motor function between AAV- and saline-treated mice using the rotarod test. **(C)** The average time of each group spent on the rotarod is indicated on the graph. The time from the starting point at week 5 in the saline-treated *Npc1*^−*/*−^ mice rapidly deteriorated, reaching 0 s at week 11. AAV-treated *Npc1*^−*/*−^ mice maintained their performance for much longer, and no mice showed a time of 0 s at week 11. However, their times gradually declined over the course. D and E show the results of statistical analyses at weeks 8 and 9, respectively. **(D)**, **(E)** The time spent on the rod was not significantly different between the AAV-treated *Npc1^+/+^* versus *Npc1*^−*/*−^, but it was significant different between the AAV-treated *Npc1*^−*/*−^ versus *Npc1*^−*/*−^ mice. ns, not significant; ***p < 0.001; **p < 0.01.

Untreated *Npc1*^−*/*−^ mice died from 63 to 86 days of age (Fig. 3A-e). Gel feeding extended the survival by roughly 20–25 days (Fig. 3A–c, d), but all untreated or saline-treated *Npc1*^−*/*−^ mice died within 13 weeks. The average survival duration of saline-treated *Npc1*^−*/*−^ mice was 98 days (range: 82–116 days) (Fig. 3A–c), whereas the average survival of AAV-*hNPC1*-treated mice was 205 days, which is 105 days longer than that of saline-treated mice. The longest survival was 310 days (Fig. 3A, b).

### Changes in the body weight

The body weight of untreated *Npc1*^−*/*−^ mice decreased progressively from week 7 (Fig. 3B, c, d, e). The AAV9/3-*hNPC1*-treated *Npc1*^−*/*−^ mice (Fig. 3B-b) weighed less than the *Npc1^+/+^* mice (Fig. 3B-a), but their body weight increased until 15 weeks of age before gradually decreasing.

### The rotarod test

Since the results of all untreated *Npc1*^−*/*−^ groups (group c, d, e) were similar, we set the saline-treated *Npc1*^−*/*−^ group as the control. In the rotarod test, the drop time of *Npc1^+/+^* mice was about 200–250 s (Fig. 3C-a). The drop time of saline-treated *Npc1*^−*/*−^ mice was already relatively short at 5 weeks, the starting point of the examination, and rapidly grew even shorter, decreasing from 120 s at 5 weeks to 0 s at 10 weeks (Fig. 3C-c). AAV-treated *Npc1*^−*/*−^ mice showed almost the same drop time as *Npc1^+/+^* mice at the beginning of the examination. The drop time of the *Npc1^+/+^* mice increased with growth. In the AAV-treated *Npc1*^−*/*−^ mice, the drop time was maintained up until 14 weeks and then gradually decreased (Fig. 3C-b). Data from weeks 8 and 9 are shown in Figs. 3D and 3E, respectively. At both points, AAV-treated *Npc1*^−*/*−^ mice showed a significantly longer drop time than saline-treated *Npc1*^−*/*−^ mice and similar drop time to *Npc1^+/+^* mice.

### Movement of mice

Untreated *Npc1*^−*/*−^ mice gradually showed difficulty in moving and eating. They showed an ataxic gait and had to keep jumping to maintain their position at weeks 8 and 9 and then showed difficulty moving smoothly and standing on their hind legs due to palsy at weeks 10 and 11. As their symptoms worsened, they became unable to remain standing on all four legs. They were unable to eat regular solid food by themselves at this point and ultimately died from weeks 10 to 12. Even in this condition, untreated *Npc1*^−*/*−^ mice were still able to eat soft gel-type food and survived to weeks 12 to 16; AAV-treated *Npc1*^−*/*−^ mice, by contrast, continued walking without problems until around week 20, and some continued to function normally up to week 35. However, AAV-treated *Npc1*^−*/*−^ mice gradually showed a spastic gait and ataxia with a wide base before their death.

### Histological analyses

**Figure 4.**
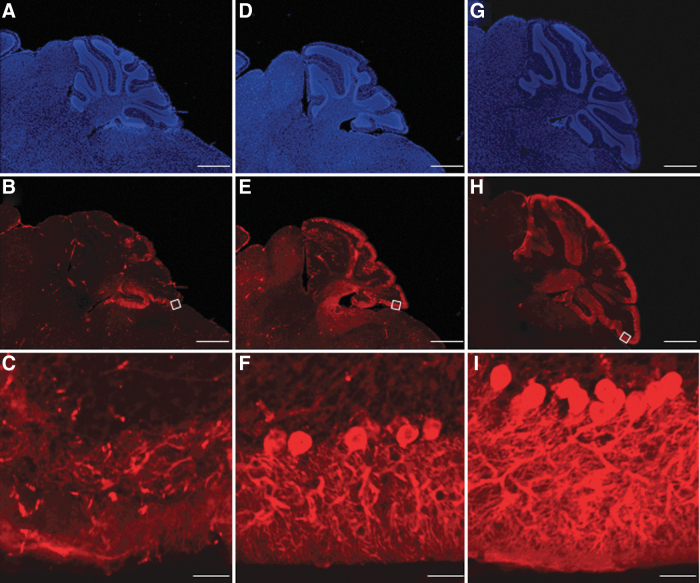
Preservation of Purkinje cells. The cerebella of untreated *Npc1*^−*/*−^ **(A–C)**, AAV-treated *Npc1*^−*/*−^ **(D–F)**, and untreated *Npc1^+/+^* mice **(G–I)** at week 11 stained with anti-calbindin antibody and Hoechst. While there were only a few Purkinje cells left in the untreated-*Npc1*^−*/*−^ mice, far more cells were observed in the AAV-treated *Npc1*^−*/*−^ mice. Scale bar = 500 μm **(A, B, D, E, G, H)**, 50 μm **(C, F, I)**.

We stained the cerebellums of untreated *Npc1^+/+^*, untreated *Npc1*^−*/*−^, and AAV-treated *Npc1*^−*/*−^ mice using an anti-calbindin antibody and Hoechst at 11 weeks of age (Fig. 4A–I). In the cerebellum, only a few Purkinje cells remained in untreated-*Npc1*^−*/*−^, whereas more Purkinje cells were maintained in AAV-treated *Npc1*^−*/*−^. In addition, granular cells and fibers were lost in untreated-*Npc1*^−*/*−^ mice, but preserved in AAV-treated *Npc1*^−*/*−^ mice. Although few neuronal cells and fibers remained in untreated *Npc1*^−*/*−^ mice, they were preserved in AAV-treated *Npc1*^−*/*−^ mice.

**Figure 5.**
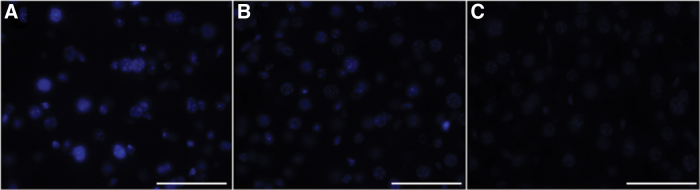
Filipin staining of each mouse liver. The accumulation of unesterified cholesterol was detected in the liver of untreated *Npc1*^−*/*−^ mice **(A)** but not detected in that of AAV-*Npc1^+/+^* mice **(C)**. The liver of AAV-*Npc1*^−*/*−^ mice **(B)** was also positive but showed a weaker signal than that of untreated *Npc1*^−*/*−^ mice. Scale bar = 50 μm **(A–C)**.

We also performed filipin staining for livers from untreated *Npc1^+/+^*, untreated *Npc1*^−*/*−^, and AAV-treated *Npc1*^−*/*−^ mice (Fig. 5A–C). The results showed the accumulation of unesterified cholesterol in untreated *Npc1*^−*/*−^ mice. Livers of AAV-treated *Npc1*^−*/*−^ mice showed weaker staining than untreated *Npc1*^−*/*−^ mice.

## DISCUSSION

We showed that the combined delivery of tyrosine-mutant AAV9/3 vectors into both the cisterna magna and lateral ventricle transduced broad areas in the CNS including the cerebellum, in a mouse model of NPC1. The survival of *Npc1*-deficient mice was markedly expanded, up by 105 days on average, and the maximum survival was 310 days after birth. The body weight was also maintained, and the rotarod performance improved and persisted for a long time. To our knowledge, these therapeutic effects are better than those described in previous studies.

It is very important to distribute the vector to broad areas of the CNS, especially the cerebellum and brain stem. Intracisterna magna (ICM) injection is reported to induce a broad distribution.^29^ The distribution is considered to be better than that after intracerebroventricle (ICV) or lumbar puncture injection.^30–32^ To achieve further distribution than ICV injection alone,^21^ we combined ICM and ICV injection. We confirmed the expression in the whole brain, including the cerebral cortex, hippocampus, cerebellum, and brain stem by combined ICM and ICV injection using AAV-GFP. The broad distribution in the brain stem and cerebral cortex was also confirmed by qPCR of the vector genome DNA, which suggests that the effects of our research are related to these functional benefits. Although the amount in the cerebellum was lower than that in other areas of the CNS, the Purkinje cells were maintained. AAV9 is well known for its ability to cross the blood/brain barrier and distribute into the brain globally, targeting both neurons and astrocytes.^33,34^ In the present study, we used the tyrosine-mutant AAV9/3 vector, which has been reported to significantly enhance gene delivery to the CNS.^24^

The lysosomal enzyme is secreted by cells and moves to other cells for integration into lysosomes to exert its effects.^35^ Therefore, many lysosomal storage diseases are treated by inducing genes in some cells in the brain, as confirmed by several studies on gene therapy for lysosomal diseases.^36^ However, as NPC1 is not secreted by cells,^37^ the *NPC1* gene needed to be delivered to a larger proportion of cells. Whether treating neurons only is sufficient or if neurons and glia both need to be treated has been unclear. NPC1 patients have been reported to show demyelination.^12^ As far as the low-density lipoprotein receptor exists without NPC1 protein, the functional loss of intracellular cholesterol trafficking will cause accumulation of cholesterol.^6^ Markedly increased levels of intracellular unesterified cholesterol in microglia have been detected since birth,^38^ as well as in various other organs in *Npc1*^−*/*−^ mice. Thus, ubiquitous promoters, such as CMV and EFα, are preferable to treat NPC1.

Thus far, three reports of NPC1 gene therapy using AAV9 vector for *Npc1*-deficient mice have been reported.^19–21^ Xie *et al.* used a CMV promoter and injected 2.5 × 10^11^ vg into the left cardiac ventricle. The average survival was extended +23 days. Chandler *et al.* used CaMK II or EF1α promoter and injected the vector into the retro-orbital venous plexus. The *Npc1*-deficient mice treated with 1.3 × 10^12^ vg using the EF1α promoter survived for 97 days longer than untreated *Npc1*^−*/*−^ mice. Hughes *et al.* used the Syn I promoter and injected 2.5 × 10^11^ vg into bilateral lateral ventricles, which extended the survival by 83 days. Systemic injection requires a substantial vector dose to transduce the CNS, so the amounts of 2.5 × 10^11^ vg reported by Xie *et al.* and 1.3 × 10^12^ vg by Chandler *et al.* might not be sufficient to obtain the maximal effect.

Of note, we injected 1.35 × 10^11^ vg in the present study, and Hughes *et al.* injected 2.5 × 10^11^ vg. However, while the injected doses were almost half, the survival time in our study was better than that reported by Hughes *et al.* One reason for this discrepancy may be because we divided the injection sites between the left ventricle and cisterna magna to achieve broader distribution of the vector. Alternatively, this difference may be because we used a universal CMV promoter that also works with glial cells, while Hughes *et al.* used the neuron-specific Syn I promoter.^39^ These points should be further analyzed.

Our treated mice survived longer than the other untreated mice, but their survival was still not as long as the natural life of mice. Although many Purkinje cells were maintained in the AAV-treated *Npc1*^−*/*−^ mice, some were lost. Therefore, to improve CNS treatment, a better method of introducing genes in all cells should be developed.

The death in untreated *Npc1*^−*/*−^ mice is considered to be due to CNS damage, since these animals showed a reduced rotarod performance and spastic palsy. The death in AAV-treated *Npc1*^−*/*−^ mice was also considered to be due to CNS damage. In patients with NPC1, CNS treatment is crucial, as the main cause of death is related to neurological dysfunction.^13^ However, multiple organ dysfunction, mainly liver dysfunction and pulmonary damage, is another possible cause of death, especially for perinatal patients who show severe liver dysfunction with cholestasis^40^ and lung damage.^41^ Our AAV-treated *Npc1*^−*/*−^ mice showed body weight loss starting a few weeks before their death, which may indicate the dysfunction of those organs. In RT-PCR, the expression of *hNPC1* was also detected in the liver, lung, and heart. The vector injected into the brain entered the blood stream and spread to the whole body. The expression of *hNPC1* in the liver and lung was lower than that in the brain, and filipin staining of the AAV-treated *Npc1*^−*/*−^ liver showed that the accumulation of unesterified cholesterol had not been entirely resolved. Based on these findings, CNS treatment may not be sufficient to treat systemic dysfunction, and adjunctive systemic injection might be required, especially for perinatal patients.

Our results are promising for the treatment of patients, although further improvements in the outcome are desired. Genomes delivered by AAV vectors are considered to be maintained for a long time in undivided cells, such as neurons. Indeed, in a nonhuman primate model of Parkinson's disease, the expression of the transgene in the brain was confirmed to persist for 15 years.^42^ Therefore, we can anticipate that treated patients will be able attain a natural life span if we can sufficiently transduce cells to maintain their function.

To achieve better outcomes, we must determine a better injection route, such as systemic injection combined with injection into the cisterna magna and bilateral ventricle. Furthermore, early diagnosis and treatment before cell death are necessary. Developing a screening system is also an important task that should be addressed in the future.

## Supplementary Material

Supplemental data

Supplemental data

Supplemental data
