# Supplementary material for: Gene Therapy in a Mouse Model of Niemann–Pick Disease Type C1
Source: Hum Gene Ther. 2021 Jun 16;32(11-12):589–98. doi: 10.1089/hum.2020.175 (PMC8236559; doi:10.1089/hum.2020.175)
Supplement: Supplemental data [file Supp_Table1.docx]

**Supplementary Table 1. Grouping of mice**

| Group | Gel feeding | Treatment | n |
| --- | --- | --- | --- |
| a AAV-treated *Npc1^+/+^* | ＋ | AAV-hNPC1 | 11 |
| b AAV-treated *Npc1^-/-^* | ＋ | AAV-hNPC1 | 12 |
| c Saline-treated *Npc1^-/-^* | ＋ | Saline | 11 |
| d Untreated *Npc1^-/-^* | ＋ | － | 6 |
| e Untreated *Npc1^-/-^* | － | － | 6 |
