# Supplementary material for: Gene Therapy in a Mouse Model of Niemann–Pick Disease Type C1
Source: Hum Gene Ther. 2021 Jun 16;32(11-12):589–98. doi: 10.1089/hum.2020.175 (PMC8236559; doi:10.1089/hum.2020.175)
Supplement: Supplemental data [file Supp_Table2.docx]

**Supplementary Table 2**. **Sequences of primers and probes to detect vector genome DNA, exogenous hNPC1, and the endogenous mNpc1 mRNA expression**

| Target | Product  Size (bp) | Primer | Annealing  Temperature (°C) |
| --- | --- | --- | --- |
| Vector (F)  (R)  (P) | 68 | 5’-GTACGGTGGGAGGTCTATATAAGCA-3’ | 60 |
|  |  | 5’-GTCGACGGATCCGAATTCG-3’ |  |
|  |  | 5’-FAM-ACCGGTTCGAAGCGA-MGB-3’ |  |
| hNPC1-1 (F)  (R) | 351 | 5’-GTGTACGGTGGGAGGTCTAT-3’  5’-TGTCTGAAGCTGCCGAACAT-3’ | 62 |
| hNPC1-2 (F)  (R) | 263 | 5’-CGGTGGGAGGTCTATATAAGCA-3’  5’-TTTGGCAATGGTTTTGGTGGG-3’ | 62 |
| mNPC1 (F)  (R) | 247 | 5’-CTACGCTGATTACCACACAC-3’  5’-AAACTCTTTCTCCCAGGCCC-3’ | 64 |
| mGAPDH (F)  (R) | 440 | 5’-CGGGGCCCACTTGAAGG-3’  5’-CCTGGAGAAACCTGCCAAGTA-3’ | 64 |

mNpc1: murine Npc1, hNPC1: human NPC1, mGAPDH: murine GAPDH,

F: forward, R: reverse, P: probe
