# Supplementary material for: Gene Therapy in a Mouse Model of Niemann–Pick Disease Type C1
Source: Hum Gene Ther. 2021 Jun 16;32(11-12):589–98. doi: 10.1089/hum.2020.175 (PMC8236559; doi:10.1089/hum.2020.175)
Supplement: Supplemental data [file Supp_Fig1.docx]

**
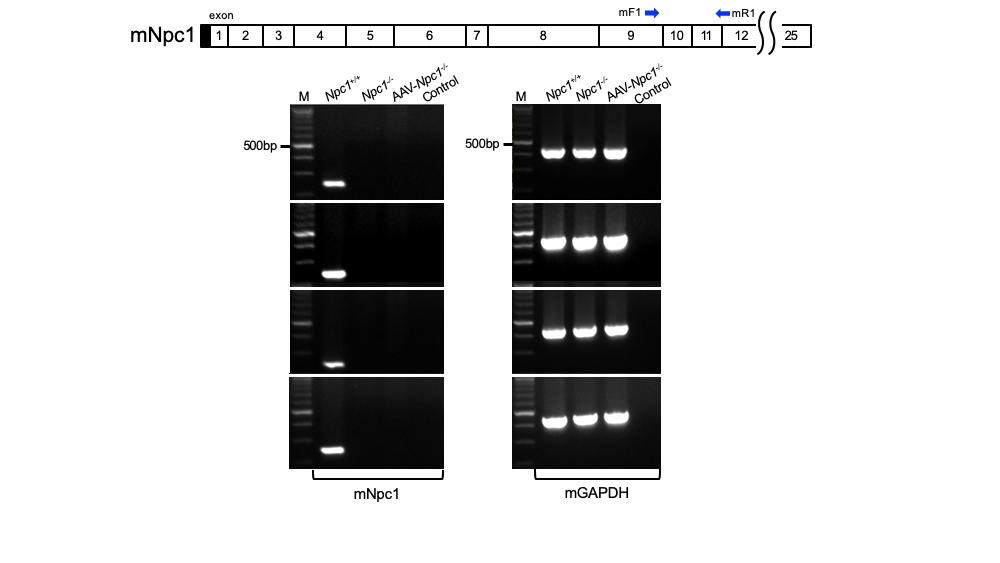
Supplementary Figure S1 legend**

Location of the primers are shown on top. The open vertical rectangles represent coding exons. The *mNpc1* mRNA was amplified using the primer set of mF1 and mR1. Detection of *mNpc1* (on the left) and mGAPDH (on the right) mRNA in the brain, liver, lung, and heart are shown in order from the top to the bottom. The expression of *mNpc1* was detected in untreated *Npc1+/+* but not in untreated nor AAV-treated *Npc1-/-* mice. mGAPDH was detected in every four tissues of all mice. Negative control didn’t show any band in both results. mNpc1, murine Niemann-Pick type C1; mGAPDH, murine glyceraldehyde-3-phosphate dehydrogenase; M, Molecular Marker; Control, Control without template cDNA; hNPC1, human Niemann-Pick type C1.
